# Postpartum histoplasmosis in an HIV-negative woman: a case report and phylogenetic characterization by internal transcribed spacer region analysis

**DOI:** 10.1590/0037-8682-0364-2019

**Published:** 2020-01-27

**Authors:** Lisandra Serra Damasceno, Antônio Mauro Barros Almeida, Bárbara de Oliveira Aguiar, Mauro de Medeiros Muniz, Marcos de Abreu Almeida, Rosely Maria Zancopé-Oliveira, Terezinha do Menino Jesus Silva Leitão

**Affiliations:** 1 Departamento de Saúde Comunitária, Faculdade de Medicina, Universidade Federal do Ceará, Fortaleza, CE, Brasil.; 2 Hospital São José de Doenças Infecciosas, Secretaria de Saúde, Fortaleza, CE, Brasil.; 3 Instituto Oswaldo Cruz, Fundação Oswaldo Cruz, Rio de Janeiro, RJ, Brasil.; 4 Instituto Nacional de Infectologia Evandro Chagas, Fundação Oswaldo Cruz, Rio de Janeiro, RJ, Brasil.

**Keywords:** Histoplasmosis, Postpartum, ITS1-5.8S-ITS2

## Abstract

The present report describes the first case of postpartum disseminated histoplasmosis in a 24-year-old HIV-negative woman. On the tenth day after vaginal delivery, the patient presented with dyspnea, fever, hypotension, tachycardia, and painful hepatomegaly. Yeast-like *Histoplasma capsulatum* features were isolated in the buffy coat. The phylogenetic analysis demonstrated that the fungal isolate was similar to other *H. capsulatum* isolates identified in HIV patients from Ceará and Latin America. Thus, histoplasmosis development in individuals with transitory immunosuppression or during the period of immunological recovery should be carefully examined.

## INTRODUCTION

Histoplasmosis is a systemic mycosis caused by *Histoplasma capsulatum*, a ubiquitous dimorphic fungus isolated from several geographic regions with distinct climates. The disease is generally asymptomatic or self-limited, although severe and disseminated infections can develop depending on the number of inhaled infective propagules, strain virulence, and host’s cellular immune response^1^. Risk factors include presence of acquired immunodeficiency syndrome (AIDS), extremes of age, immunosuppressive drug usage, hematologic malignancies, solid organ transplantation, pregnancy, and immune reconstitution syndrome (IRS)^1^. 

Few cases of disseminated histoplasmosis (DH) have been reported during pregnancy, especially in the second and third trimesters, in women with diabetes mellitus or HIV positivity^2^, but not in the postpartum period.

Here we aimed to describe the first case of DH during the postpartum period in an HIV-negative woman from an endemic area of the Brazilian Northeast and conduct a molecular analysis of the isolated *H. capsulatum*. 

## CASE REPORT

A 24-year-old woman, who was previously healthy and lived in urban area of Fortaleza, developed high fever and dry cough intensification on day 2 post vaginal delivery. The neonate was born healthy. On day 10, she was admitted to the São José Hospital of Infectious Diseases in Fortaleza, Ceará. Upon arrival at the emergency room, she was pale, dyspneic (30 breaths/min), febrile (39°C), hypotensive (blood pressure 100×60 mmHg), and tachycardic (113 beats/min). She had no lymphadenopathy and skin or oral lesions but complained of an uncommon cough that developed 2 months predelivery. Heart rhythm was normal, and pulmonary examination revealed fine crackles in the lower 2/3 of the left hemithorax. The abdomen was distended and flaccid, with painful hepatomegaly, but there was no splenomegaly.

Laboratory examinations revealed a hemoglobin level of 10 g/dL, white blood cell (WBC) count of 6,860/mm^3^ (84% neutrophils, 5% eosinophils, 10% lymphocytes, 1% monocytes), and platelet count of 321,000/mm^3^. Renal function was normal (creatinine level, 0.7, and urea level, 33 mg/dL) although lactate dehydrogenase levels were elevated (2,125 U/L). The aspartate aminotransferase level was high (67 U/L), while the alanine aminotransferase level was within normal limits (28 U/L). The arterial blood gas measurement, with a 21% fraction of inhaled oxygen, was as follows: pH=7.48, PO_2_=73.7 mmHg, PCO_2_=23.7 mmHg, and HCO_3_=17.3 mmol/L.

**FIGURE 1:**
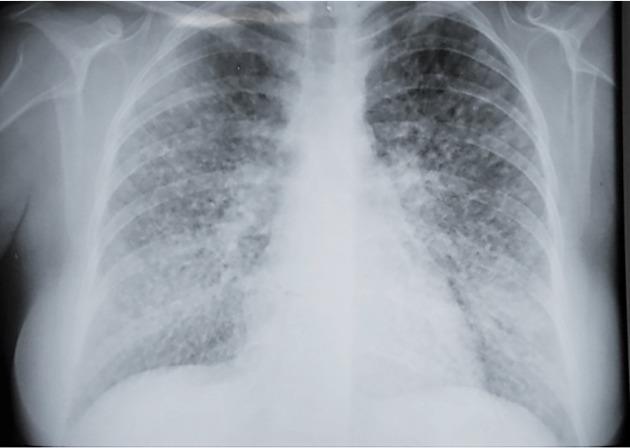
Chest X-ray showing a diffuse reticulonodular infiltrate and hilar lymphadenomegaly.

Chest radiography revealed a diffuse reticulonodular infiltrate and hilar lymphadenomegaly (Figure 1), and abdominal ultrasound showed hepatosplenomegaly and thick intrauterine liquid. HIV serology was negative.

Treatment with antituberculosis drugs, oseltamivir, levofloxacin, and dexamethasone had been initiated, although fever and dyspnea persisted, with the onset of erythematous macular pruritic skin lesions distributed in the torso and limbs, compatible with pharmacodermia. Skin biopsy was performed. Antituberculosis drug therapy was interrupted. On day 7 post-hospitalization, she developed respiratory and heart failure with refractory septic shock. Hemogram revealed a hemoglobin level of 10.6 g/dL, WBC count of 24,890/mm^3^ (93% neutrophils, 4% lymphocytes, 3% monocytes), and platelet count of 212,000/mm^3^. She was transferred to the intensive care unit, and placed on mechanical ventilation. She was then administered vasoactive drugs, and alternative treatment for tuberculosis with ethambutol, moxifloxacin, linezolid was reintroduced. In addition, piperacillin with tazobactam was initiated.

Buffy coat and respiratory sample cultures were performed. On day 12 post-hospitalization, yeast-like structures suggestive of *H. capsulatum* were found in the peripheral blood and later isolated in buffy coat culture. Culture and sputum smears for acid-fast bacilli were negative. Therapy with amphotericin B deoxycholate was initiated. Skin biopsy showed nonspecific chronic dermatitis with absence of microorganisms. Other laboratory analyses, such as C3, C4, and CH50 complement, rheumatoid factor, antineutrophil cytoplasm, antinuclear factor, and cryoglobulins, were all negative. A subsequent epidemiological link to a renovation in the patient’s home (30 days before the delivery) was obtained. After antifungal therapy, the patient showed slow and gradual improvement. She was discharged 58 days post-hospitalization with the use of itraconazole 400 mg/day, which was maintained for a year until full clinical recovery.

### Molecular aspects

*H. capsulatum* DNA was extracted as previously described^3^. The internal transcribed spacer (ITS1-5.8S-ITS2) region of the rDNA was amplified by polymerase chain reaction (PCR) using sense primer ITS5 and antisense primer ITS4, as previously described^3^. The amplicon was purified using the QIAquick PCR purification Kit (Qiagen AG, Basel, Switzerland). Automated sequencing was performed using the Sequencing Platform at the Oswaldo Cruz Foundation, PDTIS/Fiocruz, Brazil, with the same primers utilized for PCR amplification. The obtained nucleotide sequence was edited and aligned with the Clustal-W program in MEGA 6.0 software, using the sequence of the H2 strain from USA (AF322377.1), available in the GenBank database, as a reference. Additionally, the consensus sequence of the CE1714 isolate was deposited in GenBank (KX756764). After analysis by BLASTn, the CE1714 isolate sequence revealed 100% similarity with the *Ajellomyces capsulatus* (*H. capsulatum* anamorph*)* CEMM 05-2-037 strain from Ceará.

**FIGURE 2:**
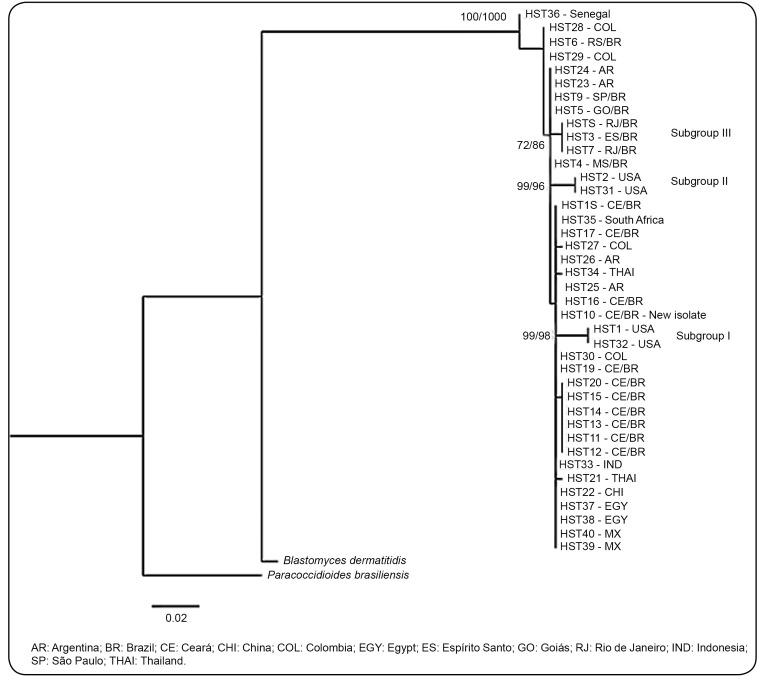
Phylogenetic tree of *H. capsulatum* isolates. The tree was constructed using the ITS1-5.8S-ITS2 region with 39 fungal sequences retrieved from GenBank and the new sequence of the CE1714 isolate. *Paracoccidioides brasiliensis* (AF322389.1) and *Blastomyces dermatitidis* (AF322389.1) were considered outgroups. The tree was generated by BI and representative of both ML and NJ analyses. The values of bt/bt are indicated in their corresponding tree nodes.

To assess the relationship of the CE1714 isolate and other *H. capsulatum* isolates from different regions, phylogenetic analysis was conducted using 39 isolates retrieved from the GenBank database (Table 1). Phylogenetic analysis was performed by maximum likelihood (ML) using the PhyML software version 3.1 and neighbor-joining (NJ) method in MEGA 6.0 software. According to the Bayesian information (BI) criterion test results, implemented in jModelTest version 2.1.6, the Kimura 81 gamma distribution model was selected. The bootstrap value (bt) analyses were based on 1000 heuristic search replicates, by estimating the alpha of the gamma parameter with four categories and empiric nucleotide frequency. The nucleotide sequence of the *Paracoccidioides brasiliensis* (AF322389.1) and *Blastomyces dermatitidis* (AF322389.1) strains were used as outgroups (Figure 2). The results showed that the CE1714 isolate presents high genetic similarity with other isolates from Latin America, Mexico, and Asia. Moreover, three specific subgroups (bt >70%) were identified in both analyses: subgroup I (HST1 and HST32 - USA), subgroup II (HST2 and HST31 - USA), and subgroup III (HST3, HST71, and HST8 - Southeast Brazil). 

**TABLE 1:** *Histoplasma capsulatum* strains and isolates used for ITS region analysis.

Strain/isolate	Name	Source	Origin	GenBank
Hc1	H2	Human/HIV+	USA	AF322377.1
Hc2	Downs	Human	USA	AF322378.1
Hc3	ES62	Human	ES-Brazil	GU320947.1
Hc4	MS53	Human	MS-Brazil	GU320981.1
Hc5	GO764	Human	GO-Brazil	GU320955.1
Hc6	157CS	Human	RS-Brazil	GU320938.1
Hc7	3237	Human	RJ-Brazil	GU320942.1
Hc8	9291	Human	RJ-Brazil	GU320940.1
Hc9	SP2414	Human	SP-Brazil	GU320951.1
**Hc10**	**CE1714**	**Human**	**CE-Brazil**	**KX756764**
Hc11	CEMM 05-2-072	Human/HIV+	CE-Brazil	JX051637
Hc12	CEMM 05-2-039	Human/HIV+	CE-Brazil	JX051639
Hc13	CEMM 05-1-098	Human/HIV+	CE-Brazil	JX051642
Hc14	CEMM 05-1-070	Human/HIV+	CE-Brazil	JX051644
Hc15	CEMM 05-1-096	Human/HIV+	CE-Brazil	JX051643
Hc16	CEMM 05-2-001	Human/HIV+	CE-Brazil	JX051647
Hc17	CEMM 05-2-034	Human/HIV+	CE-Brazil	JX051641
Hc18	CEMM 05-2-037	Human/HIV+	CE-Brazil	JX051634
Hc19	CEMM 05-2-002	Human/HIV+	CE-Brazil	JX051638
Hc20	JIEF	Human	CE-Brazil	GU320956.1
Hc21	HP12	Human/HIV+	Thailand	AB055240.2
Hc22	HP177	Human	China	AB055237.2
Hc23	HC28	Human/HIV+	Argentina	KC693532
Hc24	HC1	Human/HIV+	Argentina	KC693507
Hc25	HC38	Human/HIV+	Argentina	KC693540
Hc26	HC47	Human/HIV+	Argentina	KC693548
Hc27	H71	Human	Colombia	AF322384.1
Hc28	H70	Human/HIV+	Colombia	AF322383.1
Hc29	H68	Human	Colombia	AF322382.1
Hc30	H62	Human	Colombia	AF322379.1
Hc31	IFM41329	Human	USA	AB055228.2
Hc32	IFM41659	Human	USA	AB055230.2
Hc33	H147	Human	Indonesia	AB055235.2
Hc34	HP3	Human/HIV+	Thailand	AB055238.2
Hc35	H143		South Africa	AB055246.2
Hc36	H147	Human	Senegal	AB055247.2
Hc37	H90	Horse	Egypt	AF322387.1
Hc38	H95	Horse	Egypt	AB055249.1
Hc39	EH383		Mexico	KP132275.1
Hc40	EH374		Mexico	KP132271.1
*Paracoccidioides brasiliensis*	Outgroup	----	----	AF322389.1
*Blastomyces dermatitidis*	Outgroup	----	----	AF322388.1

**ES:** Espírito Santo; **MS:** Mato Grosso do Sul; **RS:** Rio Grande do Sul; **RJ:** Rio de Janeiro; **GO:** Goiás; **SP:** São Paulo; **CE:** Ceará.

## DISCUSSION

This report describes the first case of DH in the postpartum period in an HIV-negative woman. Phylogenetic analysis demonstrated that the fungal isolate was similar to other *H. capsulatum* clinical isolates identified in patients from Ceará and Latin America.

During pregnancy, a relative immunosuppressive state develops, characterized by decreased Th1-type response, leading to a Th2-type response that promotes fetal antigen tolerance^4^ ^,^ ^5^. Moreover, local immunoreactivity at the maternal-fetal interface also shifts toward Th2 response^4^. In this scenario, exposure to the fungal pathogen could more frequently induce the appearance of disseminated fungal infections^2^. 

In contrast, near or at delivery and in the postpartum period, recovery of Th1-type inflammatory responses occurs by broad immune activation, which is identified by increased regulatory T-cell and cytokine levels during these periods^4^ ^,^ ^5^. Some studies have suggested a significantly higher regulatory T-cell level (including CD4+ and CD8+ T lymphocytes) at delivery, which decreases with the onset of the postpartum period^6^ ^,^ ^7^. These changes can vary according to the type of delivery, maternal atopic status, and number of previous births^5^ ^,^ ^6^.

The immune response during the postpartum period constituted a complex and controversial event, given that the intense and exacerbated inflammatory response could be associated with the emergence of IRS^2^ ^,^ ^8^. Usually, IRS develops due to high microorganism or antigen loads in an unfavorable anatomic location. The diagnosis of IRS is based on the unmasking of occult asymptomatic infection or paradoxical worsening of clinical symptoms of an infection in course, unexplainably and despite appropriate antimicrobial therapy^8^. 

Thus, changes in immune response near or at delivery and shortly after the onset of the postpartum period can possibly trigger the exacerbation of several infections. In this case, specifically, the histoplasmosis may have developed due to the interaction of transitory immunosuppression of pregnancy and immunological recovery during the postpartum period. IRS development in the postpartum period has been observed concomitantly with infections, such as HIV, tuberculosis, leprosy, cryptococcosis, coccidioidomycosis, and viral hepatitis^8^. Differentiating histoplasmosis from tuberculosis may be difficult due to similarities between the two^9^. It is important to highlight the lack of availability of fast and specific methods, such as antigen detection, in the diagnosis of DH in many endemic areas, including Brazil, thus delaying the diagnosis and treatment in this regions. 

In the present study, we observed that the CE1714 isolate was clearly different compared with the USA strains but genetically similar to fungal isolates from Latin America, Mexico, and Asia. Additionally, Goldani et al. showed that fungal isolates obtained from skin lesions of patients with histoplasmosis from Rio Grande do Sul (Brazil) presented significant genetic similarity to other isolates from Colombia, Argentina, and Asia, although different from the *H. capsulatum* strain from North America^10^.

The ITS1-5.8S-ITS2 region is an excellent DNA barcode to identify diverse fungal species. This molecular region demonstrates considerable genetic diversity in *Histoplasma* spp.^11^Other studies conducted in Brazil also showed different genetic profiles among *Histoplasma* strains in America, suggesting specific microniches of the fungus in endemic areas^3^ ^,^ ^12^. The capacity to adapt to various and distinct environments and migratory flux of bats and individuals with *Histoplasma* infection have contributed significantly to this issue. 

Therefore, although severe cases of histoplasmosis occur mainly in patients with AIDS, the healthcare system should be alert regarding the development of histoplasmosis in individuals with transitory immunosuppression or those undergoing immunological recovery, such as postpartum women. 
